# Impact of 2 years of treatment with elexacaftor/tezacaftor/ivacaftor on longitudinal changes in structural lung disease in people with cystic fibrosis: results from the RECOVER trial

**DOI:** 10.1093/annalsats/aaoag021

**Published:** 2026-02-02

**Authors:** Paul McNally, Karen Lester, Rachel Cregan, Basil Elnazir, Thara Persaud, David Rea, Eilish Twomey, Mike Williamson, Des W Cox, Barry Linnane, Siobhan McGrane, Laura Kirwan, Paul O’Regan, Merlijn Bonte, Punit Makani, Daan Caudri, Jonathan D Dodd, Edward F McKone, Clare Saunders, Thomas Semple, Jane C Davies, Harm A W M Tiddens, the RECOVER Study Group, Colin Lewis, Aisling McQuaid, Aideen Lambe, David Reidy, Jennifer Matthews, Kevin Brannigan, Elizabeth Shaw, Jorien van de Puttelaar, Aleksandra Kusiak, Ava Harte, Sarah Badar, Brenda Grogan, Magdalena Mulligan, Mary Abkir, Chris Short, Tatenda Nyatoro, Benat Broderick, Caroline Heffernan, Carolyn Thornton

**Affiliations:** Department of Paediatrics, RCSI University of Medicine and Health Sciences, Dublin, Ireland; Departments of Respiratory Medicine and Radiology, Children’s Health Ireland, Dublin, Ireland; Department of Paediatrics, RCSI University of Medicine and Health Sciences, Dublin, Ireland; Departments of Respiratory Medicine and Radiology, Children’s Health Ireland, Dublin, Ireland; Department of Paediatrics, RCSI University of Medicine and Health Sciences, Dublin, Ireland; Departments of Respiratory Medicine and Radiology, Children’s Health Ireland, Dublin, Ireland; Departments of Respiratory Medicine and Radiology, Children’s Health Ireland, Dublin, Ireland; School of Medicine, Trinity College, Dublin, Ireland; Departments of Respiratory Medicine and Radiology, Children’s Health Ireland, Dublin, Ireland; Departments of Respiratory Medicine and Radiology, Children’s Health Ireland, Dublin, Ireland; Departments of Respiratory Medicine and Radiology, Children’s Health Ireland, Dublin, Ireland; Departments of Respiratory Medicine and Radiology, Children’s Health Ireland, Dublin, Ireland; Departments of Respiratory Medicine and Radiology, Children’s Health Ireland, Dublin, Ireland; School of Medicine, University College Dublin, Dublin, Ireland; School of Medicine, University of Limerick, Limerick, Ireland; Department of Paediatrics and Radiology, Limerick University Hospital, Limerick, Ireland; School of Medicine, University of Limerick, Limerick, Ireland; Department of Paediatrics and Radiology, Limerick University Hospital, Limerick, Ireland; Cystic Fibrosis Registry of Ireland, Dublin, Ireland; Cystic Fibrosis Registry of Ireland, Dublin, Ireland; Departments of Paediatric Respiratory Medicine and Radiology, Erasmus Medical Centre, Rotterdam, Netherlands; Departments of Paediatric Respiratory Medicine and Radiology, Erasmus Medical Centre, Rotterdam, Netherlands; Departments of Paediatric Respiratory Medicine and Radiology, Erasmus Medical Centre, Rotterdam, Netherlands; School of Medicine, University College Dublin, Dublin, Ireland; Departments of Respiratory Medicine and Radiology, St Vincent’s University Hospital, Dublin, Ireland; School of Medicine, University College Dublin, Dublin, Ireland; Departments of Respiratory Medicine and Radiology, St Vincent’s University Hospital, Dublin, Ireland; Department of Paediatric Respiratory Medicine, Royal Brompton & Harefield Hospitals, London, United Kingdom; National Heart and Lung Institute, Imperial College London, London, United Kingdom; Department of Paediatric Respiratory Medicine, Royal Brompton & Harefield Hospitals, London, United Kingdom; National Heart and Lung Institute, Imperial College London, London, United Kingdom; Department of Paediatric Respiratory Medicine, Royal Brompton & Harefield Hospitals, London, United Kingdom; National Heart and Lung Institute, Imperial College London, London, United Kingdom; Departments of Paediatric Respiratory Medicine and Radiology, Erasmus Medical Centre, Rotterdam, Netherlands; Thirona, Nijmegen, Netherlands

**Keywords:** cystic fibrosis, Elexacaftor/Tezacaftor/Ivacaftor, Computerised Tomography, Bronchiectasis, Automated scoring

## Abstract

**Rationale:**

Progressive structural lung disease (SLD) is a key hallmark of cystic fibrosis (CF). Elexacaftor/tezacaftor/ivacaftor (ETI) is associated with short-term improvements in SLD measured on chest computed tomography (CT). Longer-term changes in SLD outcomes with ETI are unknown.

**Objectives:**

Using multicenter standardized image collection and automated analysis, we sought to establish whether improvements in SLD with ETI continued in the second year of treatment.

**Methods:**

Spirometry-controlled CT scans were performed at 6 sites in people with CF aged ≥12 years homozygous for the F508del mutation or heterozygous for the F508del and a minimum function mutation at baseline and 12 months and 24 months after commencing ETI. CT scans were analyzed using the automated LungQ platform (Thirona) to measure mucus plugging, trapped air, and bronchus-artery (BA) pair measurements: bronchial outer diameter (B_out_), bronchial inner diameter (B_in_), bronchial wall thickness (B_wt_), arterial diameter (A), and BA ratios: B_out_/A, B_in_/A, B_wt_/A, and bronchial wall area/bronchial outer area (B_wa_/B_oa_). Scans were also visually scored using the PRAGMA-CF scoring system, divided into % disease (%DIS), % bronchiectasis (%BX), % mucus plugging (%MP), % bronchial wall thickening (%BWT), and % trapped air (%TA).

**Results:**

CT scans were performed on 79 participants at baseline, 64 at 12 months, and 52 at 24 months. Automated analysis showed a significant reduction B_wt_A, and B_wa_/B_oa_ at 12 months, which were sustained to 24 months. No change was seen in B_in_/A or B_out_/A at 12 or 24 months. Improvements in mucus plug numbers, plug volume, and %TA at 12 months were sustained at 24 months. ETI was associated with a reduction in the ratio of pulmonary blood volume in the arterial system compared to the venous system in vessels of <1 mm, 1-2 mm, and >2 mm diameter. Manual PRAGMA-CF scores demonstrated improvements in %DIS, %BX, %MP, %BWT, and %TA at 12 months, and these changes were sustained, unchanged to 24 months.

**Conclusions:**

ETI is associated with substantial improvements in bronchial wall thickening, mucus plugging, and trapped air at 12 months. Changes were maintained to 24 months and did not continue to improve. Abnormal bronchial widening remained stable and did not improve with ETI therapy. Changes in the distribution of pulmonary arterial/venous blood volumes with ETI suggest a beneficial impact on pulmonary vascular pressures.

## Introduction

Cystic fibrosis (CF) is caused by mutations in the cystic fibrosis transmembrane conductance regulator (*CFTR*) gene. The CFTR modulator combination elexacaftor/tezacaftor/ivacaftor (ETI) has been associated with significant improvements in lung function, sweat chloride, and nutrition in people with CF with at least one copy of the F508del mutation in clinical trials^1-3^ and postmarketing studies to date.^4^^,^^5^ Commercial trials of ETI have not examined outcomes relating to structural lung disease (SLD); however, postmarketing studies, including our own, have addressed this,^6-10^ using different, visual scoring systems. Most have demonstrated improvements using semi-quantitative scores in mucus plugging, bronchial wall thickening, and trapped air, but with disagreement on changes in bronchiectasis scores. Furthermore, studies have demonstrated improvements in semi-quantitative magnetic resonance imaging (MRI) scores with ETI, showing improvements in overall MRI lung scores and scores for bronchial wall thickening, mucus plugging, perfusion, and ventilation.^11-15^ MRI is widely regarded as not having sufficient resolution currently to accurately measure changes in bronchial dimensions including bronchial widening. Controversy has existed about the potential for at least partial reversibility of the bronchial widening component of bronchiectasis on computed tomography (CT), with the use of CFTR modulator therapy with a case series^16^ and prospective studies using visual^8^ and automated^17^ analyses suggesting improvements in visualized airways not seen in other studies.^6^^,^^9^^,^^10^ A recently published report in a large cohort of adolescents with CF demonstrated significant improvements in bronchial widening over 12 months of ETI therapy.^18^ Data published to date deal mostly with analysis of “before and after” imaging in the short term and does not address sequential changes in imaging over time or durability of the effect seen.

Bronchiectasis is a key pathological entity in the natural history of CF, representing progressive, seemingly irreversible injury that contributes to poor quality of life, respiratory failure, and eventually end-stage lung disease. Bronchiectasis describes abnormal widening of the bronchial diameter relative to the adjacent artery, and failure of the airway to taper as it travels to the lung periphery.^19^ The reproducibility of detection and reporting of bronchiectasis is hampered by the variability in image acquisition, processing, and visual expert-based interpretation at an individual center level.

The other important pathological entities that chest CT imaging detects in CF are mucus plugs and low attenuation regions (LARs), often referred to as trapped air. The visual assessment of mucus plugs in CF can be performed using scoring systems such as Perth Rotterdam Annotated Grid Morphometric Analysis (PRAGMA-CF), but this is technically challenging as illustrated by variable reproducibility between and even within observers.^20^ LARs, often defined as trapped air, can be observed on chest CT scans as early as infancy and gradually progress throughout life, making up an important component in end-stage lung disease.^21^^,^^22^ On expiratory scans, these LARs are likely to represent a mix of trapped air and hypoperfusion, the latter being the result of hypoxic pulmonary vasoconstriction.^23^

In order to remove human subjectivity and improve the sensitivity and accuracy of the image analysis of chest CTs, a fully automatic, artificial intelligence (AI)–based image analysis platform (LungQ, Thirona) was developed to analyze bronchus-artery (BA) dimensions of all visible BA pairs in the first to sixth subsegmental airway generations on CT scans. The algorithm also allows assessment of the number and volume of mucus plugs, areas of low attenuation, and the distribution of blood volumes in pulmonary arteries and veins. This system has been used to evaluate a number of interventions in children and adults with CF.^6^^,^^20^^,^^24^^,^^25^

RECOVER (NCT04602468) is a multicenter, phase 4 trial designed to examine the impact of ETI on a range of clinically relevant outcome measures in a real-world setting in Ireland and the United Kingdom. The primary outcome measures are lung clearance index (LCI), measured during nitrogen multiple breath washout, and forced expiratory volume in 1 second (FEV_1_). Secondary endpoints include spirometry-controlled CT scores, spirometry outcomes, sweat chloride, and nutritional and quality of life scores. We previously documented improvements in pulmonary function and visual CT scores, among other variables, at 1 year of ETI treatment in this population.^6^ For the current study, we sought to determine the longitudinal progression of SLD in this group over the second year of treatment and employ automated chest CT analysis at all timepoints, in addition to the visual approach. We hypothesized that changes in SLD seen with ETI over 1 year would remain stable in the second year on treatment and that, using automated methods to measure airway diameter, no improvement in abnormal airway widening would be seen.

To test our hypothesis, we carried out automated analysis of multicenter standardized, spirometry-controlled CT images at baseline and for 2 years after ETI initiation to measure BA dimensions and ratios, mucus plugs, trapped air, and pulmonary arterial and venous blood volume distribution. In addition, we compared visual CT scores at 24 months with previously published 12-month data and compared the outcomes generated by the automated analysis to visual PRAGMA-CF metrics and relevant physiological outcomes. Some of the results of this study were previously published in abstract form.^26^

## Methods

Participants homozygous for the F508del mutation (F508del/F508del) or heterozygous for the F508del mutation and a minimal function mutation (F508del/MF) were recruited to the study prior to ETI being initiated as part of usual care. Data on baseline characteristics, and longitudinal data on nutrition and spirometry outcomes, collected every 3 months, were taken from medical notes. Adherence to ETI was measured via medication possession ratios from pharmacy records.

Spirometry-controlled chest CT scans were performed at baseline, 12 months, and 24 months in a subgroup of study participants consenting to CT scanning as part of the study. All staff involved in conducting spirometry-controlled CT scans were trained and certified by the European Cystic Fibrosis Society Clinical Trials Network lung imaging core facility (LungAnalysis), at Erasmus Medical Centre, Rotterdam, the Netherlands. The spirometry-controlled chest CT scanning protocol used the NDD EasyOne portable spirometer (NDD Medical Technologies), as outlined previously.^15^ CT scans were pseudonymized and sent to LungAnalysis for image analysis. Manual PRAGMA-CF scoring was performed as previously described.^6^ The total effective radiation dose for this study, based on the Volume Computed Tomography Dose Index for a patient weighing 65 kg, is approximately 1.6 mSv. The radiation risk of the RECOVER study corresponds with the associated risk of approximately 7-9 months of background radiation exposure in children and young adults (the exact number depends on the patient’s age when scanned).

Automated analysis of CT scans was performed using the AI-based image analysis platform LungQ (Thirona, Nijmegen, the Netherlands) of which the senior author is an employee. BA dimensions and ratios were measured with the LungQ-BA algorithm, which automatically segments the bronchial tree, identifies the segmental bronchi (G_0_) and distal airway generations (G_1,_ G_2,_ G_3,_ etc), identifies for each airway the adjacent artery, and quantifies the following dimensions: diameters of bronchus ­outer edge (B_out_), bronchus inner edge (B_in_), and artery (A), and computes bronchus wall thickness (B_wt_). From these BA dimensions, the median BA ratios for G_1-6_ are computed: B_out_/A, B_in_/A, B_wt_/A, and bronchus wall area/bronchus outer area (B_wa_/B_oa_). The G_1_ through G_6_ range has been shown to be the optimal range of generations to monitor changes over time based on sensitivity analysis in previous cohorts. This range of generations has shown to minimize sampling bias from variable lung volumes, body size, and disease heterogeneity. To define bronchial widening, a cut-off value of B_out_/A >1.1 is applied, and a conservative cut-off value of 1.5 is used for visually apparent bronchial widening.^19^ For bronchial thickening, a cut-off value of B_wt_/A >0.14 is used.^27^ The results are given as median (range) unless otherwise stated. LungQ could not be performed on 3 participants due to missing individual slices and/or inconsistent spacing between slices.

For the analysis of mucus plugs (MPs), LungQ-MP was used. This algorithm first segments the bronchial tree and identifies each bronchopulmonary segment. Mucus plugs are detected throughout the lung and linked to their respective segments. The algorithm counts the number of mucus plugs and the total volume of mucus plugs detected in each scan. The volume of LARs was assessed using the LungQ-VERA algorithm to analyze the inspiratory and expiratory CT scans. Results are expressed as percent trapped air (%TA) of total inspiratory lung volume as computed from the inspiratory CT scan. LungQ-VERA uses voxel-wise inspiratory-expiratory image registration and global lobar volume changes to classify and quantify regions with disproportionally low lung density changes. This generates figures for the percentage of lung tissue showing trapped air (compared to normal lung).

Artery-vein phenotyping analysis (AVX) was performed on all CTs using the LungQ-AVX algorithm. LungQ-AVX quantifies the volume of each pulmonary artery (A) and vein (V) down to a diameter of 0.2 mm based on the vessel outer wall diameter and length. Next, it quantifies arterial and venous volume for arteries and veins with diameter 0.2-1 mm, 1-2 mm, and >2 mm. Blood volumes are expressed as percentages of total lung blood volume.

LCI, spirometry, sweat chloride, and Cystic Fibrosis Questionnaire–Revised Respiratory Domain scores were performed as previously described.^6^

## Statistical analysis

Baseline characteristics were summarized using median with interquartile range for continuous data, and frequency with percentage for discrete data. The F508del/F508del and F508del/MF groups were compared at baseline using Wilcoxon signed-rank tests and χ^2^ tests as appropriate. Outcome measures were analyzed using generalized linear mixed models, with an autoregressive structure for repeated measurements.^6^ Models included effects for genotype group, visit, and their interaction and their effects were tested using type III tests of fixed effects (F-tests). The number of visits varied depending on the outcome measure, but all available visit data were included in each analysis. Linear contrasts were constructed to compare baseline with all follow-up and results are presented as the difference (Δ) with 95% confidence interval and *P* value.^6^ To assess relationships among the outcome variables, Spearman correlation matrices were estimated for both static values and difference in variables between baseline and 24 months. These are presented as correlation heatmaps, with the significance of individual correlations indicated.

The study was sponsored by the RCSI University of Medicine and Health Sciences and approved by the relevant regional and local research ethics committees. All study assessments were conducted after full informed consent and assent as appropriate. Members of the CF community, supported by CF Ireland and the CF Trust, participated in the project from design through to data analysis.

## Results

### Demographics

**Figure 1:**
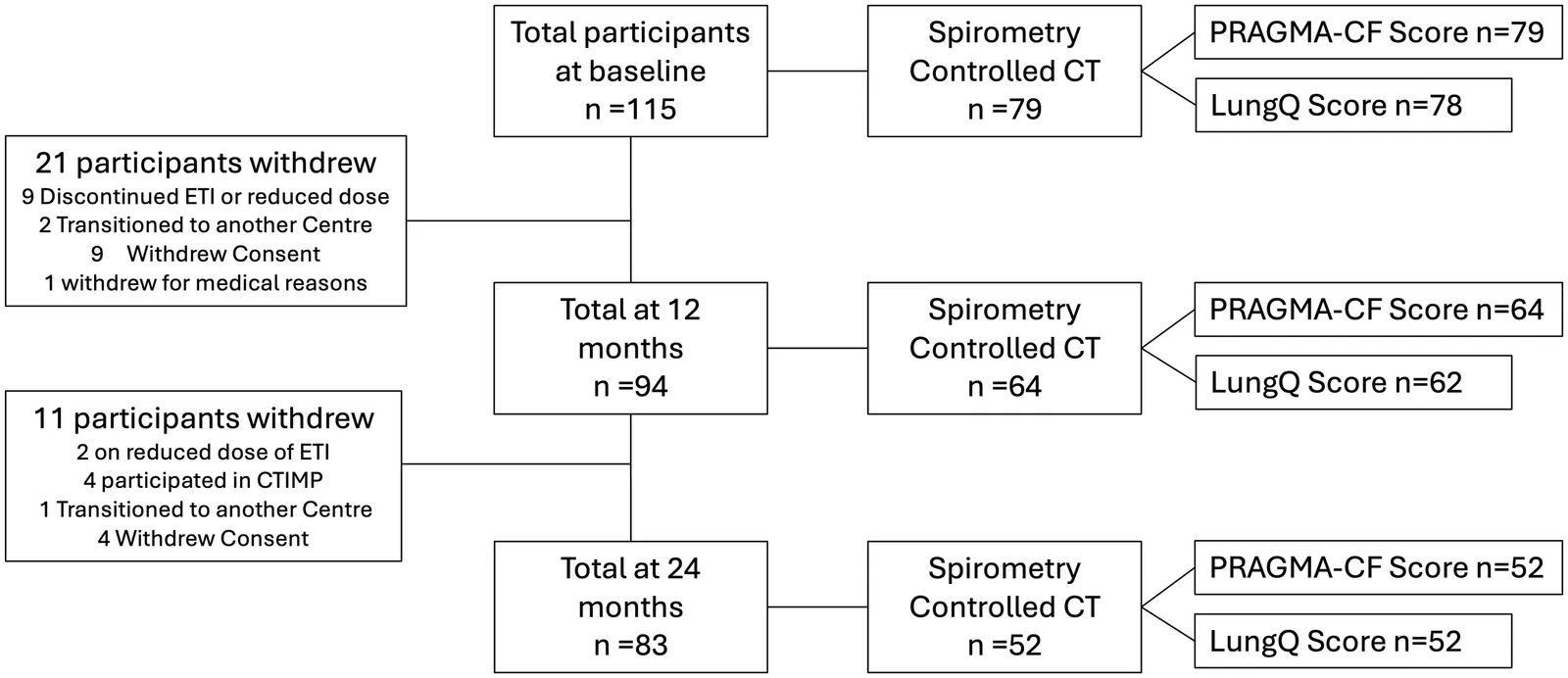
Participant flow diagram outlining retention of participants through 24 months of the study, the number of those participants undergoing CT, and the number with successfully completed CT scores. Abbreviations: CT, computed tomography; CTIMP, Clinical Trial of an Investigational Medicinal Product; ETI, elexacaftor/tezacaftor/ivacaftor; PRAGMA-CF, Perth Rotterdam Annotated Grid Morphometric Analysis.

A total of 115 participants (77 F508del/F508del, 38 F508del/MF) aged ≥12 years were recruited to the RECOVER study from 7 sites in Ireland and the United Kingdom and contributed baseline data; 97 participants remained in the study at 12 months and 83 at 24 months. A participant flow diagram (Figure 1) outlines the number of RECOVER participants per year and the number having CT imaging. Baseline characteristics of participants are outlined in Table 1. There was no difference in baseline demographics between those completing and not completing 12 or 24 months of follow-up. All but one of the F508del/F508del participants were already receiving a dual combination CFTR modulator at baseline, whereas none of the F508del/MF group were. Adherence to ETI (determined using medication possession ratio) was high in the first 12 months at 92.6%, reducing in the second 12 months to 81.2% (*P* = .007).

**Table 1:** Baseline characteristics of the study population.

Characteristic	All (*N* = 79)		Homozygous (*n* = 49)		Heterozygous (*n* = 30)		Genotype *P* value
**Age, y, median (IQR)**	14	(12, 21)	14	(12, 19)	16	(13, 22)	.141
**BMI z-score, median (IQR)**	0.23	(-0.36, 0.73)	0.15	(-0.36, 0.84)	0.33	(-0.36, 0.58)	.766
**FEV_1_pp, median (IQR)**	84.0	(74.4, 95.2)	82.3	(74.6, 93.3)	85.5	(73.2, 95.2)	.976
**LCI, median (IQR)**	11.3	(8.9, 13.7)	9.7	(8.7, 12.1)	12.3	(10.2, 15.0)	.022
**Sweat chloride, median (IQR)**	88	(72, 101)	76.7	(66, 94)	98	(89, 106)	<.0001
**Sex**							
** Female**	36	(45.6%)	21	(42.9%)	15	(50.0%)	.536
** Male**	43	(54.4%)	28	(57.1%)	15	(50.0%)	
**Age**							
** 12-17 y**	54	(68.4%)	36	(73.5%)	18	(60.0%)	.212
** ≥18 y**	25	(31.6%)	13	(26.5%)	12	(40.0%)	
***P aeruginosa* status^a^**							
** Chronic**	22	(27.8%)	11	(22.4%)	11	(36.7%)	.066
** Intermittent**	11	(13.9%)	4	(8.2%)	7	(23.3%)	
** Free**	35	(44.3%)	26	(53.1%)	9	(30.0%)	
** Never**	11	(13.9%)	8	(16.3%)	3	(10.0%)	
**FEV_1_pp <90**	52	(65.8%)	32	(65.3%)	20	(66.7%)	.902
**FEV_1_pp ≥90**	27	(34.2%)	17	(34.7%)	10	(33.3%)	
**Dornase alfa**	62	(78.5%)	37	(75.5%)	25	(83.3%)	.506
**Hypertonic saline**	63	(79.7%)	39	(79.6%)	24	(80.0%)	.741
**Pancreatic enzymes**	75	(94.9%)	48	(98.0%)	27	(90.0%)	.107
**Insulin**	9	(11.4%)	6	(12.2%)	3	(10.0%)	.761
**Oral antibiotics**	43	(54.4%)	25	(51.0%)	18	(60.0%)	.494
**Inhaled antibiotics**	29	(36.7%)	15	(30.6%)	14	(46.7%)	.151
**CFTR modulators**	48	(60.8%)	48	(98.0%)	0	(0.0%)	<.0001

Data are presented as No. (%) unless otherwise indicated. *P* values for continuous variables using Kruskal–Wallis test and *P* values for categorical variables using χ^2^ test.

Abbreviations: BMI, body mass index; CFTR, cystic fibrosis transmembrane conductance regulator; FEV_1_pp, forced expiratory volume in 1 second, percent predicted; IQR, interquartile range; LCI, lung clearance index.

a *Pseudomonas aeruginosa* status determined by Leeds criteria.

### Outcome measures

**Figure 2:**
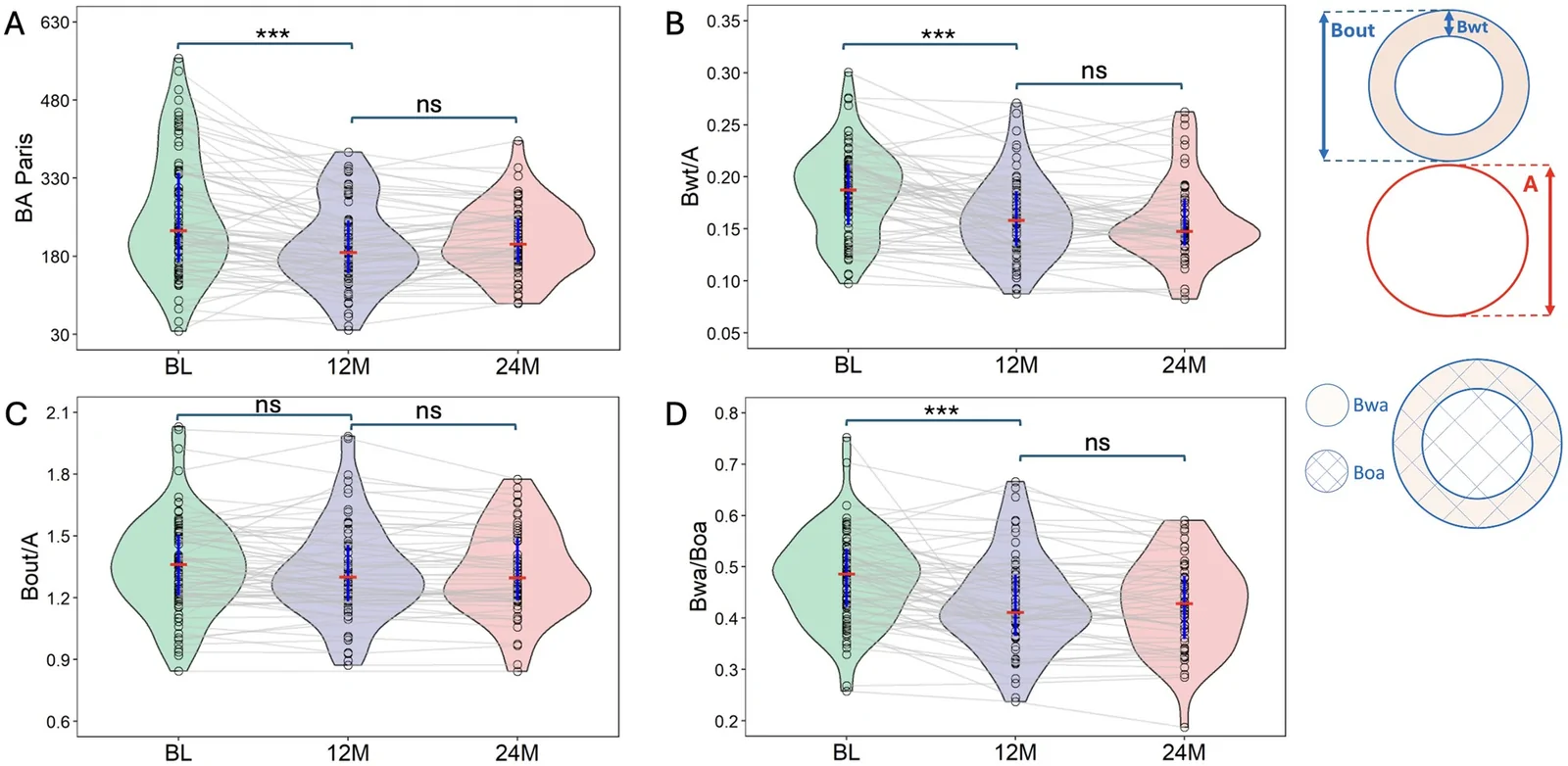
LungQ bronchus-artery (BA) analysis at baseline (BL) and at 12 months (12M) and 24 months (24M) of elexacaftor/tezacaftor/ivacaftor treatment. (A) BA pairs detected by the LungQ algorithm. (B) Ratio of bronchial wall thickening to airway diameter (B_wt_/A). (C) Ratio of outer bronchus diameter to airway diameter (B_out_/A). (D) Ratio of bronchial wall area to outer area of bronchus [lumen and wall], (B_wa_/B_oa_). ***P <.001; ns, not significant. Horizontal red line represents the median and vertical blue line represents interquartile range.

Spirometry-controlled CT scans were successfully carried out at baseline, 12 months, and 24 months on 79, 64, and 52 participants, respectively. The automated BA analysis demonstrated a significant reduction in the number of detectable BA pairs at 12 months (all mean [SE]) (259 [11.91] to 206 [12.36]; *P* < .001), which was sustained at 24 months (Table 2). There was no change in B_out_/A ratios between baseline and 12 months (1.34 [0.032] to 1.30 [0.035]; *P* = .263) or between 12 and 24 months (1.30 [0.035] to 1.33 [0.039]; *P* = .415). Similarly, B_in_/A did not demonstrate any change with ETI (Table 2, Figure 2). B_wt_/A improved significantly at 12 months (0.19 [0.005] to 0.16 [0.006]; *P* < .001) and was sustained at 24 months (Table 2). B_wa_/B_oa_ improved significantly at 12 months (0.48 [0.012] to 0.42 [0.013]; *P* < .001) and was sustained at 24 months (Table 2, Figure 2).

**Table 2:** Summary of LungQ scores over 24 months of treatment with elexacaftor/tezacaftor/ivacaftor in people homozygous and heterozygous for the F508del mutation.

**All participants**	Baseline (*N* = 78)		12 mo (*n* = 64)		24 mo (*n* = 52)		Baseline vs 12 mo		12 mo vs 24 mo		*P* values for tests of fixed effects		
	Mean	SE	Mean	SE	Mean	SE	Δ (95% CI)	*P* value	Δ (95% CI)	*P* value	Time	Genotype	Time * genotype
**BA pairs**	259.0	11.91	206.3	12.36	219.5	13.50	-52.6 (-69.7, -35.6)	<.001	13.2 (-6.6, 32.9)	.189	<.001	.141	.854
**B_out_/A**	1.34	0.032	1.30	0.035	1.33	0.039	-0.04 (-0.1, 0.03)	.263	0.03 (-0.04, 0.1)	.415	.450	.031	.603
**B_in_/A**	0.95	0.028	0.97	0.030	1.00	0.033	0.03 (-0.02, 0.08)	.306	0.02 (-0.03, 0.08)	.398	.340	.097	.516
**B_wt_/A**	0.19	0.005	0.16	0.006	0.16	0.006	-0.03 (-0.04, -0.02)	<.001	0 (-0.01, 0.01)	.823	<.001	.041	.402
**B_wa_/B_oa_**	0.48	0.012	0.42	0.013	0.43	0.015	-0.06 (-0.09, -0.04)	<.001	0.01 (-0.02, 0.04)	.485	<.001	.830	.129
**Plug count**	7.2	1.92	0.52	0.146	0.65	0.183	0.07 (0.06, 0.09)	<.001	1.26 (1, 1.59)	.048	<.001	.016	<.001
**Plug volume**	3.22	0.394	0.13	0.433	0.23	0.498	-3.09 (-4.19, -1.99)	<.001	0.11 (-1.14, 1.36)	.866	<.001	.104	.134
**%TA**	14.8	0.91	8.2	0.98	8.3	1.08	-6.6 (-8.4, -4.8)	<.001	0.14 (-1.96, 2.23)	.897	<.001	.046	.352

**F508del/F508del**	Baseline (*n* = 48)		12 mo (*n* = 39)		24 mo (*n* = 35)		Baseline vs 12 mo		12 mo vs 24 mo	
	Mean	SE	Mean	SE	Mean	SE	Δ (95% CI)	*P* value	Δ (95% CI)	*P* value
**BA pairs**	239.2	14.71	191.3	15.28	204.8	16.03	-47.9 (-69.2, -26.5)	<.001	13.4 (-9.6, 36.4)	.251
**B_out_/A**	1.30	0.040	1.24	0.043	1.25	0.045	-0.06 (-0.14, 0.02)	.142	0.01 (-0.08, 0.1)	.806
**B_in_/A**	0.93	0.035	0.93	0.037	0.94	0.039	0 (-0.06, 0.06)	.998	0.02 (-0.05, 0.08)	.651
**B_wt_/A**	0.18	0.006	0.15	0.007	0.15	0.007	-0.02 (-0.04, -0.01)	.001	-0.01 (-0.02, 0.01)	.417
**B_wa_/B_oa_**	0.47	0.015	0.43	0.016	0.42	0.017	-0.04 (-0.07, -0.01)	.023	-0.01 (-0.04, 0.03)	.640
**Plug count**	4.4	1.50	0.26	0.095	0.28	0.103	0.06 (0.04, 0.08)	<.001	1.09 (0.75, 1.58)	.662
**Plug volume**	2.11	0.488	0.05	0.541	0.05	0.570	-2.06 (-3.44, -0.69)	.004	0 (-1.49, 1.48)	.996
**%TA**	12.4	1.13	7.1	1.21	6.9	1.26	-5.3 (-7.6, -3.0)	<.001	-0.21 (-2.69, 2.27)	.867

**F508del/MF**	Baseline (*n* = 30)		12 mo (*n* = 25)		24 mo (*n* = 20)		Baseline vs 12 mo		12 mo vs 24 mo	
	Mean	SE	Mean	SE	Mean	SE	Δ (95% CI)	*P* value	Δ (95% CI)	*P* value
**BA pairs**	278.7	18.72	221.3	19.44	234.2	21.73	-57.4 (-84.1, -30.7)	<.001	12.9 (-19.1, 44.9)	.426
**B_out_/A**	1.38	0.051	1.37	0.054	1.42	0.063	-0.01 (-0.11, 0.09)	.796	0.05 (-0.07, 0.17)	.407
**B_in_/A**	0.97	0.044	1.02	0.046	1.05	0.054	0.05 (-0.03, 0.13)	.190	0.03 (-0.06, 0.13)	.474
**B_wt_/A**	0.20	0.008	0.16	0.009	0.17	0.010	-0.03 (-0.05, -0.02)	<.001	0.01 (-0.01, 0.03)	.386
**B_wa_/B_oa_**	0.49	0.019	0.40	0.020	0.43	0.024	-0.09 (-0.13, -0.05)	<.001	0.03 (-0.02, 0.08)	.229
**Plug count**	11.5	4.82	1.03	0.440	1.50	0.643	0.09 (0.07, 0.11)	<.001	1.47 (1.12, 1.92)	.006
**Plug volume**	4.32	0.618	0.20	0.676	0.42	0.817	-4.12 (-5.85, -2.4)	<.001	0.22 (-1.79, 2.23)	.830
**%TA**	17.2	1.43	9.3	1.53	9.7	1.74	-7.9 (-10.8, -5.0)	<.001	0.48 (-2.89, 3.85)	.776

Plug count has been modeled using a generalized linear mixed model with a Poisson distribution and a log link. The predicted means and SEs have been back-transformed to the count scale. The 95% CIs display the ratios of the comparisons, eg, 0.07 (0.06-0.09) should be interpreted as a 93% reduction in plugs between baseline and 12 months; 1.26 (1.0-1.59) implies a 26% increase in plugs between 12 months and 24 months. All other response variables have not been transformed and assume residuals are normally distributed. *P* values for tests of fixed effects of time, genotype group, and the product of both in the model are shown for the whole group.

Abbreviations: BA, bronchus-artery; B_in_/A, ratio of bronchial inner diameter to artery; B_oa_, bronchial outer area; B_out_/A, ratio of bronchial outer diameter to artery; B_wa_, bronchial wall area; B_wt_/A, ration of bronchial wall thickening to artery; CI, confidence interval; MF, minimum function; SE, standard error; %TA, percent trapped air.

**Figure 3:**
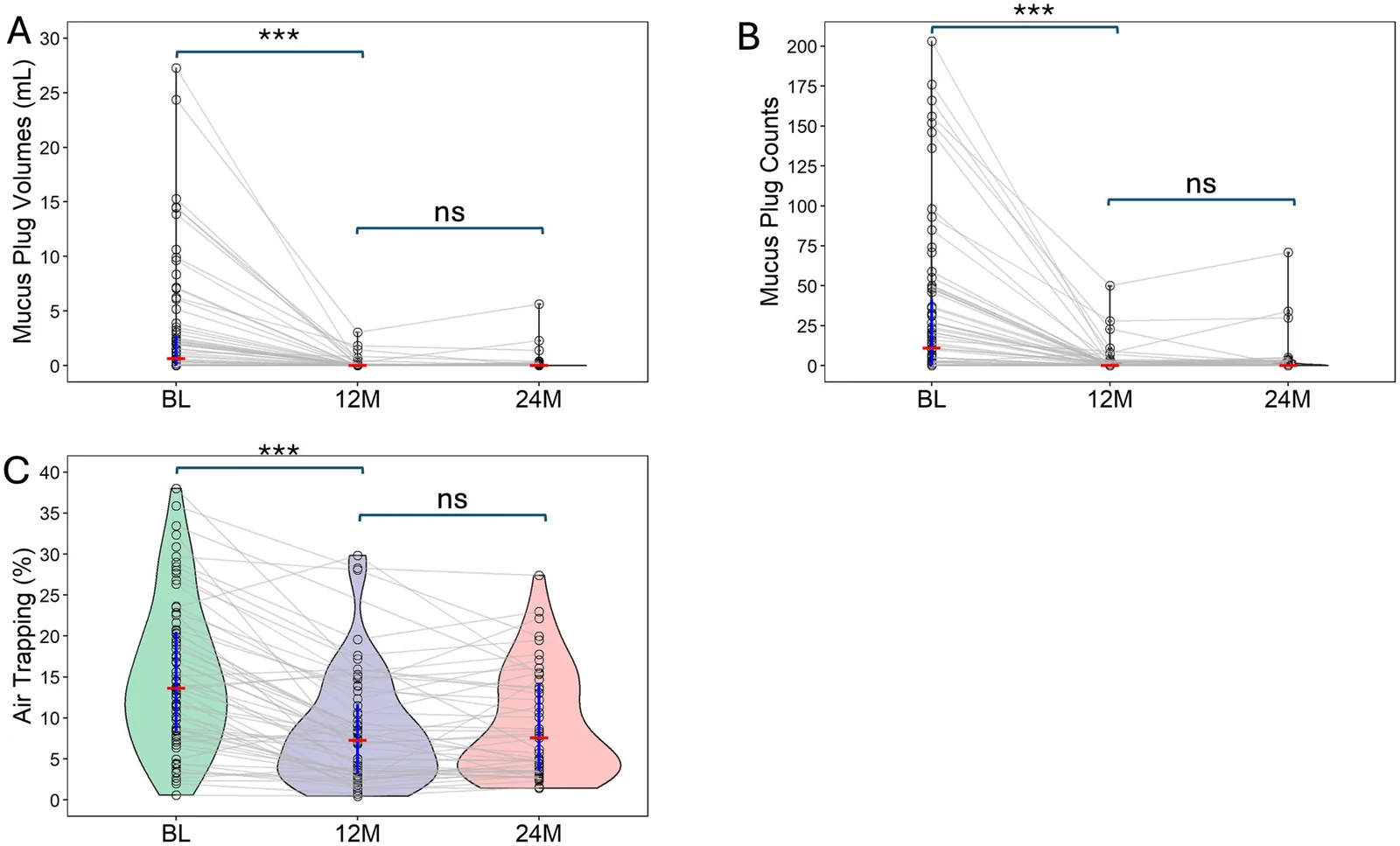
LungQ analysis of mucus plugging and trapped air at baseline (BL) and at 12 months (12M) and 24 months (24M) of elexacaftor/tezacaftor/ivacaftor treatment. (A) Mucus plug volumes (mL). (B) Mucus plug counts. (C) Percentage trapped air using the VERA algorithm. ***P <.001; ns, not significant. Horizontal red line represents the median and vertical blue line represents interquartile range.

**Figure 4:**
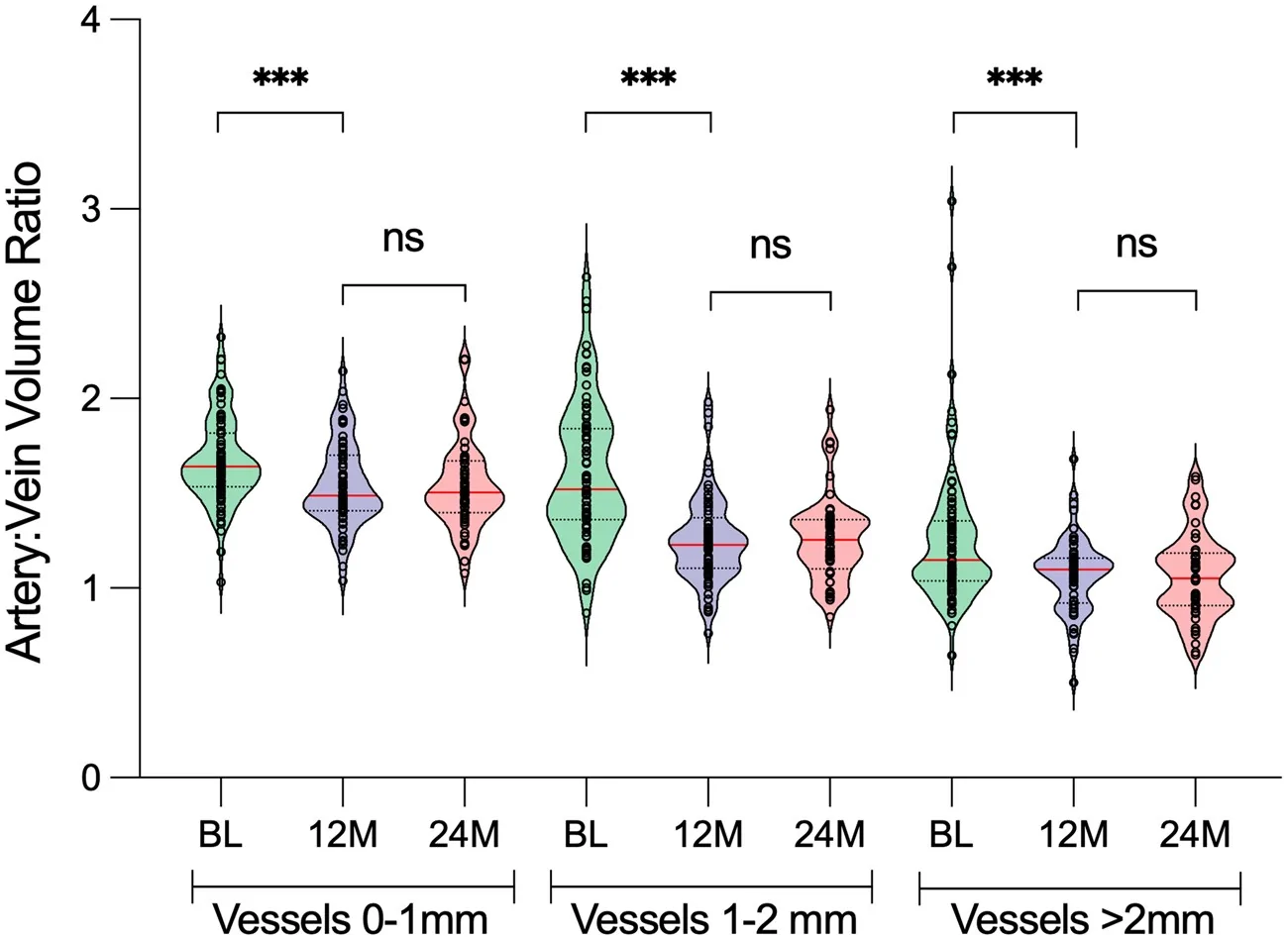
LungQ artery-vein phenotyping analysis blood volume distribution. Ratio of blood volumes in arterial and venous vessels at baseline (BL), 12 months (12M), and 24 months (24M) in vessels of sizes 0-1 mm, 1-2 mm, and >2 mm. ***P <.001; ns, not significant. Horizontal red lines indicate median and dashed lines the interquartile range.

Using the LungQ-MP algorithm, both the (log transformed) number (7.2 [1.92] to 0.52 [0.146]; *P* < .001) and volume (3.22 [0.394] to 0.13 [0.433]; *P* < .001) of mucus plugs reduced significantly at 12 months and was sustained at 24 months (Table 3). Similarly, using the LungQ-VERA algorithm, the %TA air as a proportion of total lung volume declined significantly at 12 months (14.8 [0.91] to 8.2 [0.98]; *P* < .001) and was sustained at 24 months (Table 2, Figure 3). The LungQ-AVX analysis showed an overall reduction of percentage blood volume in medium-sized and large pulmonary arteries and an increase in medium-sized and large pulmonary veins as outlined in Table 3. ETI was associated with a reduction in the ratio of pulmonary blood volume in the arterial system compared to the venous system (A:V) in vessels of 0.2-1 mm, 1-2 mm, and >2 mm diameter (1.67 [0.035] to 1.50 [0.038], 1.62 [0.039] to 1.22 [0.042], and 1.27 [0.036] to 1.02 [0.040], all *P* < .001; Table 4, Figure 4).

**Table 3:** LungQ analysis of percentage of blood volume in pulmonary arteries and veins of size 0-1 mm, 1-2 mm, and >2 mm and ratios of blood volume in arterial to venous vessels of the same sizes.

Characteristic	All Participants															
	Baseline (*N* = 79)			12 mo (*n* = 71)			24 mo (*n* = 61)			Baseline vs 12 mo		12 mo vs 24 mo		*P* values for tests of fixed effects		
	*N*	Mean	SE	*N*	Mean	SE	*N*	Mean	SE	Δ (95% CI)	*P* value	Δ (95% CI)	*P* value	Time	Genotype	Time * genotype
**Arteries 0-1 mm**	78	6.72	0.183	64	6.52	0.198	52	6.80	0.222	-0.2 (-0.62, 0.22)	.351	0.27 (-0.2, 0.75)	.259	.476	.405	.394
**Veins 0-1 mm**	78	4.03	0.105	64	4.21	0.114	52	4.40	0.128	0.18 (-0.07, 0.42)	.166	0.2 (-0.09, 0.48)	.168	.029	.976	.540
**Arteries 1-2 mm**	78	10.94	0.241	64	9.44	0.262	52	9.91	0.297	-1.5 (-2.09, -0.91)	<.001	0.47 (-0.2, 1.14)	.168	<.001	.133	.893
**Veins 1-2 mm**	78	6.89	0.156	64	7.55	0.171	52	7.80	0.197	0.66 (0.22, 1.1)	.003	0.25 (-0.25, 0.74)	.320	<.001	.824	.496
**Arteries >2 mm**	78	38.91	0.781	64	35.20	0.848	52	36.05	0.955	-3.7 (-5.55, -1.86)	<.001	0.85 (-1.25, 2.94)	.424	<.001	.525	.080
**Veins >2 mm**	78	31.46	0.661	64	33.81	0.726	52	34.98	0.835	2.35 (0.52, 4.18)	.012	1.17 (-0.9, 3.24)	.265	.002	.188	.748
**A:V 0-1 mm**	78	1.67	0.035	64	1.50	0.038	52	1.56	0.043	-0.16 (-0.25, -0.08)	<.001	0.05 (-0.04, 0.15)	.245	.001	.148	.913
**A:V 1-2 mm**	78	1.62	0.039	64	1.22	0.042	52	1.28	0.047	-0.4 (-0.48, -0.31)	<.001	0.06 (-0.04, 0.16)	.227	<.001	.105	.136
**A:V >2 mm**	78	1.27	0.036	64	1.02	0.040	52	1.05	0.044	-0.25 (-0.33, -0.16)	<.001	0.03 (-0.07, 0.12)	.554	<.001	.151	.029

	F508del/F508del												
	Baseline (*n* = 49)			12 mo (*n* = 46)			24 mo (*n* = 41)			Baseline vs 12 mo		12 mo vs 24 mo	
	*N*	Mean	SE	*N*	Mean	SE	*N*	Mean	SE	Δ (95% CI)	*P* value	Δ (95% CI)	*P* value
**Arteries 0-1 mm**	48	6.75	0.227	39	6.27	0.247	35	6.64	0.258	-0.48 (-1.01, 0.04)	0.070	0.37 (-0.2, 0.93)	.198
**Veins 0-1 mm**	48	4.11	0.130	39	4.19	0.142	35	4.33	0.148	0.09 (-0.23, 0.4)	0.588	0.14 (-0.2, 0.47)	.415
**Arteries 1-2 mm**	48	10.72	0.298	39	9.15	0.327	35	9.53	0.343	-1.57 (-2.3, -0.83)	<0.001	0.38 (-0.42, 1.18)	.348
**Veins 1-2 mm**	48	7.05	0.193	39	7.60	0.214	35	7.67	0.226	0.55 (0.01, 1.1)	0.047	0.07 (-0.52, 0.66)	.811
**Arteries >2 mm**	48	37.27	0.968	39	35.52	1.057	35	36.15	1.105	-1.75 (-4.06, 0.56)	0.135	0.63 (-1.85, 3.12)	.616
**Veins >2 mm**	48	32.00	0.819	39	34.86	0.907	35	35.28	0.956	2.86 (0.58, 5.14)	0.014	0.41 (-2.05, 2.87)	.741
**A:V 0-1 mm**	48	1.62	0.043	39	1.45	0.047	35	1.53	0.050	-0.17 (-0.27, -0.07)	0.001	0.07 (-0.04, 0.18)	.182
**A:V 1-2 mm**	48	1.51	0.049	39	1.1963	0.053	35	1.25	0.055	-0.31 (-0.42, -0.21)	<0.001	0.05 (-0.06, 0.17)	.355
**A:V >2 mm**	48	1.16	0.045	39	1.01	0.049	35	1.04	0.051	-0.15 (-0.25, -0.04)	0.0068	0.03 (-0.09, 0.14)	.627

	F508del/MF												
	Baseline (*n* = 30)			12 mo (*n* = 25)			24 mo (*n* = 20)			Baseline vs 12 mo		12 mo vs 24 mo	
	*N*	Mean	SE	*N*	Mean	SE	*N*	Mean	SE	Δ (95% CI)	*P* value	Δ (95% CI)	*P* value
**Arteries 0-1 mm**	30	6.69	0.287	25	6.78	0.310	17	6.95	0.362	0.09 (-0.57, 0.74)	.794	0.18 (-0.59, 0.94)	.650
**Veins 0-1 mm**	30	3.95	0.164	25	4.22	0.178	17	4.48	0.209	0.27 (-0.12, 0.66)	.180	0.26 (-0.2, 0.71)	.266
**Arteries 1-2 mm**	30	11.16	0.378	25	9.72	0.410	17	10.28	0.486	-1.44 (-2.36, -0.51)	.003	0.56 (-0.52, 1.64)	.306
**Veins 1-2 mm**	30	6.74	0.245	25	7.50	0.268	17	7.93	0.324	0.77 (0.08, 1.45)	.028	0.43 (-0.37, 1.22)	.289
**Arteries >2 mm**	30	40.54	1.226	25	34.89	1.326	17	35.96	1.558	-5.65 (-8.53, -2.77)	<.001	1.07 (-2.31, 4.44)	.532
**Veins >2 mm**	30	30.92	1.036	25	32.76	1.134	17	34.69	1.369	1.84 (-1.02, 4.69)	.204	1.93 (-1.4, 5.26)	.254
**A:V 0-1 mm**	30	1.71	0.055	25	1.55	0.060	17	1.59	0.070	-0.16 (-0.29, -0.03)	.016	0.03 (-0.11, 0.18)	.644
**A:V 1-2 mm**	30	1.72	0.062	25	1.25	0.067	17	1.31	0.077	-0.48 (-0.61, -0.34)	<.001	0.07 (-0.09, 0.23)	.411
**A:V >2 mm**	30	1.38	0.057	25	1.03	0.062	17	1.06	0.072	-0.35 (-0.48, -0.22)	<.001	0.03 (-0.13, 0.18)	.706

Data are shown for all participants and those of the different genotype groups at baseline, 12 months, and 24 months. *P* values for tests of fixed effects of time, genotype group, and the product of both in the model are shown for the whole group.

Abbreviations: A:V, ratio of blood volume in arterial to venous vessels; CI, confidence interval; MF, minimum function; SE, standard error.

**Table 4:** PRAGMA-CF scores for % disease (overall score), % bronchiectasis, % bronchial wall thickening, % mucus plugging, and % trapped air in all participants, those with the F508del/F508del genotype, and those with the F508del/minimum function genotype at baseline, 12 months, and 24 months.

**Characteristic**	All Participants															
	Baseline (*N* = 79)			12 mo (*n* = 71)			24 mo (*n* = 61)			Baseline vs 12 mo		12 mo vs 24 mo		*P* values for tests of fixed effects		
	*N*	Mean	SE	*N*	Mean	SE	*N*	Mean	SE	Δ (95% CI)	*P* value	Δ (95% CI)	*P* value	Time	Genotype	Time * genotype
**% Disease**	78	7.42	0.702	64	4.04	0.415	52	4.47	0.445	-3.61 (-4.4, -2.8)	<.001	0.54 (-0.4, 1.5)	.263	<.001	.056	.020
**% Bronchiectasis**	78	4.10	0.431	64	3.29	0.392	52	3.41	0.385	-0.79 (-1.3, -0.3)	.001	0.19 (-0.4, 0.8)	.506	.005	.141	.664
**% Mucus plugging**	78	2.10	0.365	64	0.10	0.041	52	0.22	0.073	-2.21 (-2.9, -1.6)	<.001	0.14 (-0.6, 0.9)	.705	<.001	.028	.009
**% Bronchial wall thickening**	78	1.17	0.115	64	0.62	0.090	52	0.81	0.117	-0.56 (-0.8, -0.3)	<.001	0.17 (-0.1, 0.4)	.218	<.001	.089	.256
**% Trapped air**	78	18.85	1.773	63	10.61	1.688	52	8.81	1.719	-9.97 (-12.9, -7.1)	<.001	-1.94 (-5.2, 1.3)	.238	<.001	.013	.005

	F508del/F508del												
	Baseline (*n* = 49)			12 mo (*n* = 46)			24 mo (*n* = 41)			Baseline vs 12 mo		12 mo vs 24 mo	
	*N*	Mean	SE	*N*	Mean	SE	*N*	Mean	SE	Δ (95% CI)	*P* value	Δ (95% CI)	*P* value
**% Disease**	48	6.07	0.768	39	3.42	0.443	35	3.88	0.457	-2.42 (-3.4, -1.4)	<.001	0.29 (-0.8, 1.4)	.608
**% Bronchiectasis**	48	3.73	0.496	39	2.82	0.409	35	2.88	0.430	-0.8 (-1.4, -0.2)	.012	-0.07 (-0.7, 0.6)	.841
**% Bronchial wall thickening**	48	0.96	0.115	39	0.51	0.080	35	0.82	0.132	-0.41 (-0.7, -0.1)	.008	0.26 (-0.1, 0.58)	.109
**% Mucus plugging**	48	1.34	0.338	39	0.08	0.034	35	0.16	0.072	-1.26 (-2.1, -0.4)	.003	0.08 (-0.8, 1)	.862
**% Trapped air**	48	13.55	1.917	39	9.13	1.745	35	7.25	1.692	-5.2 (-8.7, -1.7)	.004	-1.78 (-5.6, 2)	.356

	F508del/MF												
	Baseline (*n* = 30)			12 mo (*n* = 25)			24 mo (*n* = 20)			Baseline vs 12 mo		12 mo vs 24 mo	
	*N*	Mean	SE	*N*	Mean	SE	*N*	Mean	SE	Δ (95% CI)	*P* value	Δ (95% CI)	*P* value
**% Disease**	30	9.60	1.269	25	5.00	0.779	17	5.67	0.937	-4.79 (-6.1, -3.5)	<.001	0.78 (-0.7, 2.3)	.312
**% Bronchiectasis**	30	4.70	0.792	25	4.03	0.761	17	4.51	0.726	-0.88 (-1.7, -0.1)	.028	0.46 (-0.5, 1.4)	.336
**% Bronchial wall thickening**	30	1.49	0.228	25	0.79	0.190	17	0.80	0.238	-0.71 (-1.1, -0.3)	<.001	0.08 (-0.4, 0.5)	.724
**% Mucus plugging**	30	3.33	0.734	25	0.14	0.090	17	0.34	0.167	-3.17 (-4.2, -2.1)	<.001	0.21 (-1, 1.4)	.732
**% Trapped air**	30	27.33	2.856	24	13.02	3.405	17	12.02	3.913	-14.73 (-19.3, -10.2)	<.001	-2.11 (-7.4, 3.2)	.429

*P* values for tests of fixed effects of time, genotype group, and the product of both in the model are shown for the whole group.

Abbreviations: CI, confidence interval; MF, minimum function; SE, standard error.

Compared to previously reported 12-month scores,^6^ no significant changes in visual PRAGMA-CF CT scores were seen at 24 months (Table 4). With the addition of the 24-month data to the mixed-effects model, and the inclusion of previously unanalyzed data, statistically significant improvements in all scores and subscores, including the bronchiectasis scores, in both the F508del/F508del and F508del/MF groups were seen through 24 months (Table 4).

At baseline, significant differences were seen in sweat chloride and LCI in those in the F508del/F508del group compared to the F508del/MF group. These persisted at 24 months (Table 5). Differences were seen at baseline between the genotype groups in LungQ parameters of volume of mucus plugs, %TA, and A:V volume ratios in medium and large vessels. These differences were not seen at 24 months; however, the F508del/MF group had a higher B_out_/A and a higher mucus plug count at 24 months. Differences were seen at baseline between the genotype groups for all PRAGMA-CF outcomes except % bronchiectasis. At 24 months, no differences could be seen in any PRAGMA-CF score between the groups.

**Table 5:** Genotype group comparisons of core outcomes at baseline and 24 months from the mixed-effects model.

**Characteristic**	Baseline									24 mo								
	F508del/F508del			F508del/MF			Difference			F508del/F508del			F508del/MF			Difference		
	*N*	Mean	SE	*N*	Mean	SE	Difference	95% CI	*P* value	*N*	Mean	SE	*N*	Mean	SE	Difference	95% CI	*P* value
**Sweat**	48	78.64	2.588	29	97.09	2.496	-18.39	(-26.4, -10.4)	**<.0001**	41	39.53	2.845	19	62.62	4.268	-23.20	(-32.4, -14)	**<.0001**
**FEV_1_pp**	49	84.50	2.011	30	82.89	2.606	1.61	(-4.7, 7.9)	.617	40	89.51	1.956	19	89.74	3.544	-1.90	(-8.6, 4.8)	.577
**LCI**	37	10.71	0.432	24	13.06	0.845	-2.36	(-4, -0.7)	**.006**	35	9.25	0.523	20	11.61	1.001	-1.94	(-3.6, -0.2)	**.027**
**PRAGMA-CF score^a^**																		
** %DIS**	48	6.07	0.768	30	9.60	1.269	-3.58	(-5.8, -1.3)	**.002**	35	3.88	0.457	17	5.67	0.937	-1.60	(-4.2, 1)	.219
** %BX**	48	3.73	0.496	30	4.70	0.792	-1.01	(-2.6, 0.6)	.209	35	2.88	0.430	17	4.51	0.726	-1.45	(-3.2, 0.3)	.104
** %BWT**	48	0.96	0.115	30	1.49	0.228	-0.53	(-0.9, -0.1)	**.012**	35	0.82	0.132	17	0.80	0.238	-0.06	(-0.6, 0.4)	.806
** %MP**	48	1.34	0.338	30	3.33	0.734	-1.99	(-2.9, -1.1)	**<.0001**	35	0.16	0.072	17	0.34	0.167	-0.17	(-1.3, 1)	.770
** %TA**	48	13.55	1.917	30	27.33	2.856	-13.66	(-19.8, -7.5)	**<.0001**	35	7.25	1.692	17	12.02	3.913	-3.65	(-11, 3.7)	.326
**LungQ**																		
** BA pairs**	48	239.60	17.683	30	278.70	12.270	-39.51	(-86.7, 7.7)	.100	35	207.66	21.893	17	221.53	14.320	-29.43	(-82.9, 24.1)	.278
** B_out_/A**	48	1.30	0.044	30	1.38	0.035	-0.079	(-0.21, 0.05)	.224	35	1.28	0.043	17	1.42	0.050	-0.165	(-0.32, -0.01)	**.037**
** B_in_/A**	48	0.93	0.037	30	0.97	0.036	-0.042	(-0.15, 0.07)	.451	35	0.96	0.038	17	1.04	0.036	-0.113	(-0.24, 0.02)	.090
** B_wt_/A**	48	0.18	0.007	30	0.20	0.006	-0.020	(-0.04, 0)	.055	35	0.15	0.008	17	0.18	0.012	-0.025	(-0.05, 0)	.051
** B_wa_/B_oa_**	48	0.46	0.016	30	0.49	0.016	-0.025	(-0.07, 0.02)	.315	35	0.42	0.016	17	0.44	0.019	-0.013	(-0.07, 0.05)	.663
** Plug count**	48	24.33	6.227	30	42.73	0.866	0.385	(0.13, 1.12)	.079	35	1.66	9.864	17	6.82	4.474	0.189	(0.06, 0.58)	**.004**
** Plug volume**	48	2.11	0.691	30	4.32	0.041	-2.212	(-3.77, -0.65)	**.006**	35	0.07	1.086	17	0.53	0.346	-0.372	(-2.35, 1.6)	.710
** %TA**	48	12.41	1.288	30	17.15	1.087	-4.740	(-8.36, -1.12)	**.011**	35	7.03	1.784	17	11.10	1.752	-2.820	(-7.08, 1.44)	.192
** A:V 0-1 mm**	48	1.62	0.048	30	1.71	0.037	-0.089	(-0.23, 0.05)	.207	35	1.54	0.045	17	1.56	0.069	-0.062	(-0.23, 0.11)	.468
** A:V 1-2 mm**	48	1.51	0.058	30	1.72	0.032	-0.213	(-0.37, -0.06)	**.008**	35	1.24	0.076	17	1.30	0.069	-0.063	(-0.25, 0.12)	.506
** A:V >2 mm**	48	1.16	0.039	30	1.38	0.034	-0.223	(-0.37, -0.08)	**.003**	35	1.06	0.096	17	1.08	0.068	-0.020	(-0.2, 0.16)	.821

Figures in bold indicate statistical significance. Abbreviations: A:V 0-1 mm, 1-2 mm, and >2 mm, ratios of blood volume in arterial to venous vessels of size 0-1 mm, 1-2 mm, and >2 mm; BA, bronchus-artery; B_in_/A, ratio of bronchus inner diameter; B_oa_, bronchial outer area; B_out_/A, ratio of bronchial outer area to artery; B_wa_, bronchial wall area; B_wt_/A, ratio of bronchial wall thickness to artery; FEV_1_pp, forced expiratory volume in 1 second, percent predicted; LCI, lung clearance index; MF, minimum function; PRAGMA-CF, Perth Rotterdam Annotated Grid Morphometric Analysis; SE, standard error.

a PRAGMA-CF score encompasses % disease (%DIS), % bronchiectasis (%BX), % bronchial wall thickening (%BWT), % mucus plugging (%MP), and % trapped air (%TA).

**Figure 5:**
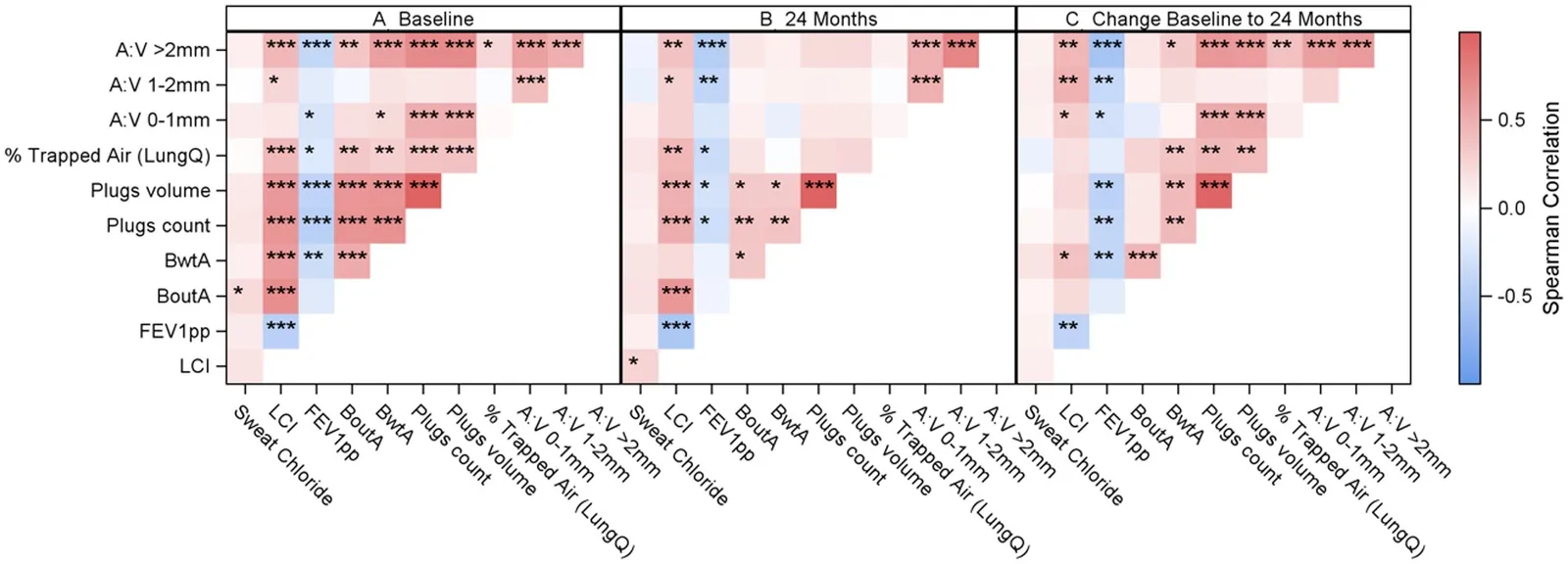
Correlation matrix of key outcome measures from LungQ analysis and clinical outcomes of sweat chloride, FEV_1_ percent predicted, and LCI. Spearman correlation was used. *P <.05, **P <.01, ***P <.001. Abbreviations: A:V, Arterial:Venous; B_out_/A, Ratio of outer bronchial diameter to artery; B_wt_/A, Ratio of bronchial wall thickness to artery; FEV_1_pp, forced expiratory volume in 1 second, precent predicted; LCI, lung clearance index.

A number of patterns can be seen in the relationship between clinical and LungQ outcomes (see specific details on strength and significance of individual correlations in Figure 5). First, there was little correlation between sweat chloride and any other outcome in this cohort. Second, lung function measures were generally well correlated to imaging outcomes, with a stronger association at baseline compared to 24 months and a stronger relationship overall between LCI and LungQ outcomes than between FEV_1_ and LungQ outcomes. Third, A:V vascular volume ratios in large vessels correlated better with clinical outcomes than ratios in smaller vessels. Last, static relationships between outcomes were stronger at baseline than at 24 months in general. We saw no relationship between improvements in sweat chloride and markers of SLD.

## Discussion

We demonstrate here, using sensitive automated image analysis of chest CT, that ETI is associated with significant improvements in SLD that are maintained for up to 2 years in people with CF. Improvements are seen in airway wall thickening, mucus plugging, and trapped air, but not in abnormal airway widening, the key airway injury in bronchiectasis. The changes that we observed in arterial and venous blood volumes also suggest an effect of ETI on pulmonary vascular pressures. The use of automated analysis has allowed us to assess with great sensitivity and accuracy changes in airway and artery dimensions and ratios of a large number of BA pairs in each subject that would not have been possible using manual visual systems.

Airway disease in CF is progressive, with chronic and persistent infection and inflammation resulting in cumulative airway damage. At any point in the disease, some elements are likely to be reversible and some irreversible. When thinking about the impact of correcting CFTR dysfunction on airway disease, it is important to establish which aspects of disease can be reversed (at least temporarily) and which cannot. This will help us to understand disease progression, the optimal timing of interventions, and how to adapt standard of therapy and monitoring. Bronchiectasis is a widely used term, but in clinical practice is poorly defined and understood. The key component of bronchiectasis is abnormal airway widening, judged on subjective visual inspection by radiologists and pulmonologists. Until recently it was not feasible to execute actual measurements of BA dimensions and ratios as this would be too time consuming. Bronchiectasis has long been considered irreversible. But as accurate and sensitive methods have been lacking, the natural history of bronchial widening and thickening and its response to therapies has remained largely unknown. Using this technology applied to control cohorts of people with CF, reference values for the progression of airway widening (using B_out_/A ratios) have been established.^27^ Based on these data, over the first year of treatment we would have expected B_out_/A to increase by 0.03 (95% CI, -0.012 to 0.072), whereas in our cohort we see a reduction of 0.036 in that period (a nonsignificant change), suggesting that ETI is at least preventing continued worsening of abnormal airway widening, but not clearly improving it. The study by Sermet-Gaudelus et al. showed a reduction in B_out_/A of 0.09 in the first year on ETI.^18^

Using manual PRAGMA-CF scores, we have shown that bronchiectasis scores improve with ETI. However, the assessment of widening is done visually and therefore only on BA pairs that are visible to the naked eye. Our data show clearly that the number of detectable BA pairs reduces with ETI treatment. The algorithm, like the naked eye, requires contrast between adjacent areas to detect anatomical structures. Airway wall thickening leads to an increase in the number of visible airways by providing this contrast to adjacent tissues.^28^ Thus, some of the improvements seen in bronchiectasis scores in our data and that of others may be explained by a lower number of abnormally thickened (and hence visualized) airways. In addition, the superior sensitivity of automated over manual scores in detecting changes in airway wall thickening and widening has previously been demonstrated in trials of both hypertonic saline^24^ and azithromycin^25^ in children, suggesting that this is a more reliable measure than visual assessment.

A French study, Modul-CF, reported significant improvements over 12 months in B_out_/A, whereas we did not see this over 24 months despite both studies demonstrating significant improvements in mucus plugging and bronchial wall thickening. The reason for this difference in bronchial widening outcomes is likely down to a number of issues: Modul-CF had a significantly greater modulator-naïve cohort (*n* = 125 in whom dramatic improvements were seen in B_out_/A: 1.41 to 1.27) compared to individuals switching from dual modulators (*n* = 63, 1.22 to 1.20), whereas our study mostly recruited people switching (*n* = 48) compared to modulator naïve (*n* = 30). Our study had smaller automated analysis numbers (*n* = 78 vs 188), included adults and adolescents, and involved spirometry control and image standardization, all of which may have affected the results. Further longitudinal data and post-hoc analyses from these and other real-world studies will be required to shed light on this discrepancy in due course.

Areas of low attenuation on expiratory imaging in CF have long been appreciated and typically referred to as “trapped air”; however, some debate surrounds this classification. We showed considerable improvement in LARs with ETI treatment with the automated and visual analyses. At baseline and 24 months, a modest correlation was seen between LARs and ventilation inhomogeneity measured by LCI, but changes over time in these variables were not correlated. In general, LARs seen on chest CT imaging could be related to either reduced blood flow or larger gas volumes in a particular area compared to surrounding lung, and some controversy exists around what LARs represent in CF. This prompted us to examine changes in blood volumes in pulmonary arteries and veins in response to ETI treatment. Our data show that ETI has a measurable impact on pulmonary blood volumes, but whether this effect is primary or secondary to improved lung health is unclear. We found little correlation, however, between trapped air and LungQ automated blood volume measurements (Figure 5). Localized airway hypoxia is well described in CF,^29^^,^^30^ and a hypoxic environment can affect pulmonary vasculature via hypoxic pulmonary vasoconstriction.^31^ Our findings are novel in CF and may represent improvement in these pathways, but a direct effect on vessels cannot be excluded.

Our data, like that of other groups, shows a significant genotype effect in the most commonly used CF physiological readout, sweat chloride, in response to ETI. People with 2 copies of F508del achieve lower sweat chloride values, with a higher likelihood of entering the normal range on treatment.^6^ Genotype differences in clinical outcomes are less commonly reported. We reported some differences in clinical outcomes responses between the F508del/F508del and F508del/MF groups after 1 year.^6^ Longitudinal data collection out to 24 months gives us the opportunity to determine if these differences are sustained or relating just to differences in short-term effects. At baseline we see significant differences in several markers of SLD, as may be expected, given that almost all in the F508del/F508del group had been on dual CFTR modulators for several years and those in the heterozygous group had been on none. By 24 months, most of the between-group differences have resolved; however, differences in B_out_/A and mucus plug count have become significant, suggesting that beneficial effects on mucus plugging and airway widening are different between the groups.

Our data provide strong evidence for the benefit of ETI on SLD in people with CF, with the observed benefit sustained for up to 2 years on treatment. However, while potentially “reversible” aspects of SLD such as bronchial wall thickening, mucus plugs, and trapped air are significantly improved overall, in many individuals they do not normalize. Furthermore, established abnormal bronchial widening does not appear to improve with therapy, in our cohort at least, and finally we do not know how long this beneficial effect on SLD will be sustained for in subsequent years. With the benefit of longer-term data on the first CFTR modulator ivacaftor, which produced similar improvements in sweat chloride, we have seen that pulmonary function improved initially but subsequently declined, albeit at a slower rate than in comparators,^32^^,^^33^ with some evidence that longitudinal rates of decline in pulmonary function on ivacaftor are lower in younger children.^34^ Longitudinal collection of real-world imaging data will therefore be essential if we are to understand how long the improvements in SLD can be sustained on ETI and how biological factors such as existing SLD, age, sweat chloride levels, and genotype groups influence rates of change.

This study has some limitations. We do not have a control group in this real-world, postapproval trial, and so we cannot say with certainty that the changes in outcomes are all caused by ETI. Most of our subjects were adolescents rather than adults, so our population may have earlier milder lung disease and different rates of progression than that of other adult cohorts. The number of participants with CT scans available for analysis reduced during the study, with only 52 available at 24 months, which may have reduced our power to detect subtle differences in outcomes. Our study commenced prior to the release of the updated Spiroware software, version 3.3.1, using a previous version that is now known to overestimate LCI values. There has been much debate over the handling of legacy data and the best correction approach.^31^ We acknowledge that the LCI_2.5_ results reported from this study will be somewhat overestimated as previously discussed,^6^ but on the basis of previous comparisons, there is likely little impact on the delta change. The LungQ-BA algorithm analyses airways from the first to sixth subsegmental bronchi. While this is more than can be analyzed visually, early CF lung disease manifests also in peripheral airways, which are not detected with this algorithm. Changes in these smaller airways result in hypoventilation and hypoperfusion, which can be detected as low attenuation areas (trapped air) on expiratory scans.

We believe that this is an important study for a number of reasons: First, we have shown that an automated system can sensitively measure and score longitudinal changes in key aspects of SLD in people with CF. Second, we have demonstrated that initial improvements in CF SLD and pulmonary vasculature with ETI are sustained, but do not further improve, in the second year on treatment. Finally, we have demonstrated that bronchial wall thickening, but not abnormal widening, improves with ETI, bringing important new information to the debate about reversibility of bronchiectasis in the CF literature.

## Supplementary Material

aaoag021_Supplementary_Data
